# Genetic Ablation of Pannexin1 Protects Retinal Neurons from Ischemic Injury

**DOI:** 10.1371/journal.pone.0031991

**Published:** 2012-02-23

**Authors:** Galina Dvoriantchikova, Dmitry Ivanov, David Barakat, Alexander Grinberg, Rong Wen, Vladlen Z. Slepak, Valery I. Shestopalov

**Affiliations:** 1 Department of Ophthalmology, Bascom Palmer Eye Institute, University of Miami Miller School of Medicine, Miami, Florida, United States of America; 2 Vavilov Institute of General Genetics, Russian Academy of Sciences, Moscow, Russian Federation; 3 National Institute of Child Health and Development, National Institutes of Health, Bethesda, Maryland, United States of America; 4 Department of Molecular Pharmacology, University of Miami Miller School of Medicine, Miami, Florida, United States of America; 5 Department of Cell Biology and Anatomy, University of Miami Miller School of Medicine, Miami, Florida, United States of America; National Institute on Aging Intramural Research Program, United States of America

## Abstract

Pannexin1 (Panx1) forms large nonselective membrane channel that is implicated in paracrine and inflammatory signaling. *In vitro* experiments suggested that Panx1 could play a key role in ischemic death of hippocampal neurons. Since retinal ganglion cells (RGCs) express high levels of Panx1 and are susceptible to ischemic induced injury, we hypothesized that Panx1 contributes to rapid and selective loss of these neurons in ischemia. To test this hypothesis, we induced experimental retinal ischemia followed by reperfusion in live animals with the Panx1 channel genetically ablated either in the entire mouse (Panx1 KO), or only in neurons using the conditional knockout (Panx1 CKO) technology. Here we report that two distinct neurotoxic processes are induced in RGCs by ischemia in the wild type mice but are inactivated in Panx1KO and Panx1 CKO animals. First, the post-ischemic permeation of RGC plasma membranes is suppressed, as assessed by dye transfer and calcium imaging assays *ex vivo* and *in vitro*. Second, the inflammasome-mediated activation of caspase-1 and the production of interleukin-1β in the Panx1 KO retinas are inhibited. Our findings indicate that post-ischemic neurotoxicity in the retina is mediated by previously uncharacterized pathways, which involve neuronal Panx1 and are intrinsic to RGCs. Thus, our work presents the *in vivo* evidence for neurotoxicity elicited by neuronal Panx1, and identifies this channel as a new therapeutic target in ischemic pathologies.

## Introduction

Neuronal ischemia caused by the loss of blood supply to the brain or retina leads to ATP depletion, followed by the inhibition of Na+/K+ pumps, the collapse of membrane potential and global ionic disregulation [1], [2]. Physiological studies have suggested ionotropic glutamate and kainate receptors [3], [4], [5], calcium channels [6], [7] and, more recently, hemichannels [8], [9] to be implicated in these pathological events. Pannexin1 protein, encoded by the *Panx1* gene, is a mammalian membrane channel-forming protein structurally and evolutionary related to invertebrate gap junction proteins [10], [11], [12]. Whereas gap junction full channels coordinate electric and metabolic activity of contacting cells via full channels, their half-channels (hemichannels) communicate the intra- and extracellular compartments and serve as a diffusional pathway for ions and small molecules [13]. Pannexins form membrane channels incapable of coupling into functional gap junctions [14], which distinguishes them from connexins [15].

The Panx1 channel has high electrical conductance and is permeable to small molecules and metabolites including ATP, IP3, LPS, NAD+, Ca^2+^, glucose, glutamate, arachidonic acid and glutathione among others [15]. This channel opens in response to membrane depolarization and increase in cytosolic Ca^2+^, while its interactions with various membrane receptors render Panx1 responsive to mechanical stimulation, extracellular purines, high extracellular K^+^, and other stimuli [11], [15], [16], [17], [18]. A more recent study showed proteolytic activation of Panx1 by caspase-3 digestion and indicated that the channel plays an essential role in phagocyte attraction during apoptosis [19]. Currently, the normal physiological function of Panx1 remains poorly understood. It was shown that cell swelling and membrane breakdown after ischemic injury are blocked by hemichannel inhibitors in pyramidal neurons, which express Panx1 but not connexins [8], [20]. These data, together with the findings that Panx1 channels are opened by extracellular ATP [17], nitric oxide [20] and glutamate [21], suggested that Panx1 activation facilitates neurotoxicity in ischemic brain [9].

Panx1 is also involved in the activation of a cytoplasmic protein complex known as the inflammasome. The inflammasome mediates proteolytic activation of caspases-1, which is a critical step in processing and secretion of pro-inflammatory cytokines IL-1β, IL-18 and IL-33 in monocytes, astrocytes, as well as brain neurons [22], [23]. Over-production of IL-1β was shown to play deleterious role in the central nervous system (CNS) [24] and inflammasome activation is now being implicated in multiple neurological conditions [25], including brain and spinal cord injury [26], [27]. The IL-1β toxicity can be suppressed by interleukin-1 receptor blockade, which alleviated damage in retinal ischemia model [28], [29], [30]. Anti-IL-1β therapy is now a clinically proven therapy of autoinflammatory diseases, familial hereditary fever, gout, rheumatoid arthritis and type 2 diabetes mellitus [31], [32], [33] and is in clinical trials for stroke patients [34]. Equally efficient neuroprotection is achieved by alternative strategy, i.e. by direct blockade of inflammasome, as shown in rodent models of stroke and traumatic brain injury [26], [27]. The exact nature of signal leading to inflammasome activation in the CNS remains poorly understood. Among the mechanisms suggested recently is Panx1 channel-mediated internalization of external danger signals [35], [36] and Panx1-mediated activation of caspase-1 [23], [37]. However, the connection between neuronal Panx1 channel and molecular underpinnings of ischemic degeneration of neurons remains to be investigated.

The overall aim of this study is to examine the role of Panx1 channels in the pathophysiology of retinal IR injury *in vivo*. The hemichannel blockers such as carbenoxolone (CBX), lanthanum and mefloquine that were commonly utilized to study the function of Panx1 in cultured cells are not suitable for this purpose. These chemicals are rather non-specific as they also inhibit connexons and stimulate unrelated pathways. To circumvent potential non-specific effects commonly associated with pharmacological inhibitors, we developed a conditional Panx1 knockout mouse. This mouse model was instrumental to study the relationship between Panx1 activity and pathophysiology of ischemic loss of RGCs in the retina. We report here that Panx1 deficiency protects RGCs from death induced by ischemia. Our data provide evidence that Panx1 is involved in at least two toxicity mechanisms and suggest that Panx1 is a convergence point for several neurotoxic pathways and a new target for therapeutic intervention in retinal ischemic disorders.

## Results

### Panx1 conditional knockout mouse line

To study the role of Panx1 channels in retinal ischemia we generated homozygous Panx1fl/fl (“floxed”) mutant mice, harboring three LoxP consensus sites inserted into a single-copy *Panx1* gene (Panx1/LoxP line, Fig. S1). Founders with germline transmission of ES cell-derived genome were heterozygous for the mutant Panx1allele and were bred for homozygocity. The resulting mice were crossed with CMV-Cre and Thy1-Cre strains to create the global KO (CMV-Cre/Panx1) and neuron-specific conditional CKO (Thy1-Cre/Panx1) knockout lines. *Cre*-mediated recombination within the *Panx1* gene resulted in a germline removal of the LoxP-flanked exons 3 and 4. These two lines were backcrossed to C57Bl/6 background for at least five generations and bred to homozygocity afterwards.

**Figure 1:**
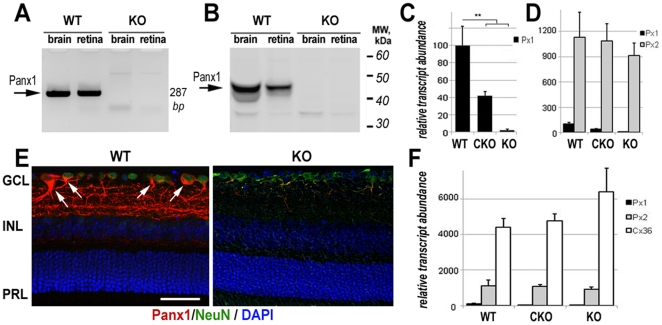
Analysis of Panx1 ablation in CMV-cre/Panx1 (KO) mouse retinas. **A**. RT-PCR analysis of retinal and brain homogenates from wild type (WT), Panx1 CKO (CKO) and Panx1 KO (KO) mice using primers for the floxed Exon 4 (287 bp band, arrow). **B**. Western blot analysis of total retina homogenates probed with rabbit anti-Panx1 CT-395(Px-34) antibodies [38] shows a 47 kDA band (arrow) corresponding to Panx1. **C**. Quantitative RT-PCR analysis of relative abundance of Panx1 transcripts in total retinal extracts from the animals of all three genotypes. **D**. Relative abundance of Panx1 and Panx2 transcripts in total extracts from WT, CKO and KO retinas. The values were normalized to the levels of *Actb* (β-actin), with the data presented as mean±SD. Double asterisk indicates *P*<0.01. **E**. Confocal micrographs of retinal sections from WT and KO mice probed with anti-Panx1 CT-395 antibodies (red) and co-labeled with RGCs marker Neuronal Class III β-Tubulin (green), and nuclei marker DAPI (blue). RGC somatae with Panx1-specific labeling are indicated by arrows. Scale bar, 25 µm. **F**. Relative abundance of Panx1, Panx2 and Cx36 transcripts the WT, CKO and KO retinas. Transcript abundances were normalized to the levels of *Actb*; data are presented as mean±SD. Double asterisk indicates *P*<0.01.

Disruption of the gene and the transcript was confirmed by Southern blot, RT-PCR and partial genome sequencing (Fig. 1A and Fig. S1). Panx1 protein ablation was validated by Western blot and immunohistochemistry (IHC) in the Panx1 KO retinas (Fig. 1B,E) using mouse monoclonal anti-Panx1 CT-395 antibodies [38], and with two other commercially available antibodies (Fig. S2). Both the conditional and global *Panx1* knockouts were fertile and indistinguishable from WT littermates by gross retinal morphology, RGC density and presence of a- and b-waves of standard flash electroretinograms (Table S1; Fig. S3).

According to quantitative RT-PCR, neuron-specific Panx1 knockout resulted in a 60% decrease in the abundance of the Panx1 transcript compared to WT retinas (Fig. 1C). This partial reduction was expected because Panx1 is also present in non-Thy-1-expressing neurons, glial and vascular endothelial cells [39], [40]. In contrast, the global Panx1 KO line lacked the transcript entirely. To test whether Panx1 deficiency causes compensatory changes in other hemichannels, we measured the expression of the pannexin2 and connexin36 genes, but no statistically significant changes were detected (Fig. 1 D,F).

### IR-induced RGC loss is suppressed by the ablation of Panx1

**Figure 2:**
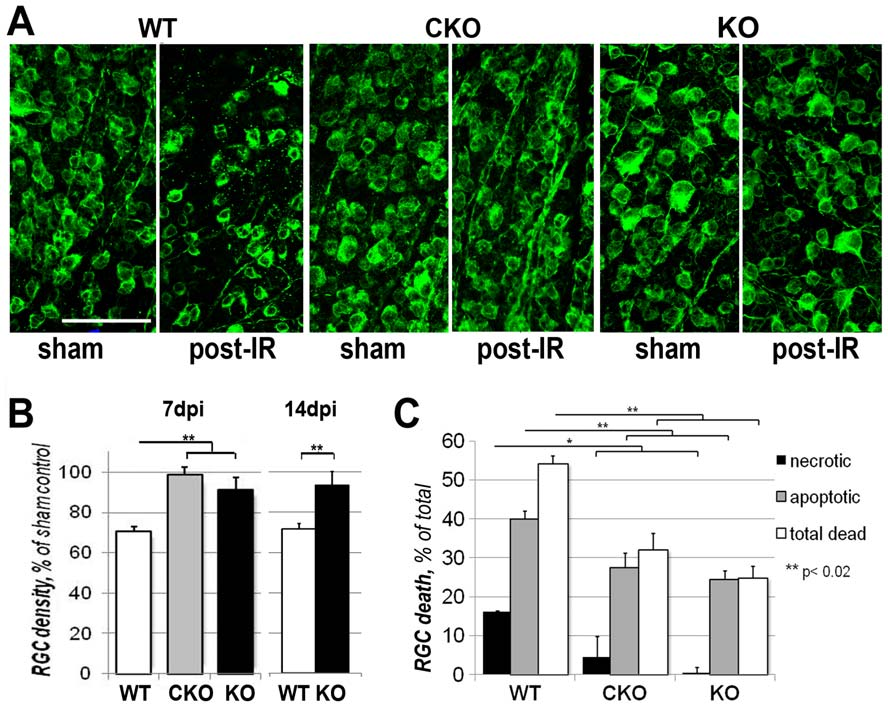
Protective effect of Panx1 KO in ischemic retina. **A**. Representative micrographs of retinas from experimental (post-IR) and control sham treated (sham) eyes from WT, Panx1 CKO (CKO) and Panx1 KO (KO) animals challenged by retinal IR. RGCs are labeled with Neuronal ClassIII β-Tubulin antibodies for counting. Scale bar, 50 µm. **B**. Neuronal survival was calculated as the percentage of cells in experimental vs. fellow sham-operated eyes (mean±SD, n = 8); RGC survival rates were measured at 7 and 14 days after IR using direct cell count in retinal flat-mounts. **C**. RGC Survival and the rates of apoptotic and necrotic cells in the OGD-challenged primary RGCs cultures from Panx1 KO and WT retinas 24 h after OGD. Annexin V/Propidium Iodide (PI) co-labeling was utilized to analyze cell death.

Several pathophysiological events of the ischemic CNS injury, including ionic disbalance, anoxic depolarization and oxidative stress can be explained by an abrupt permeation of neuronal plasma membrane [8], [20]. Therefore, we hypothesized that a pathological cascade leading to ischemia-induced RGC loss is triggered by the opening of Panx1 channels. To test whether this model is correct, we compared the extent of RGC loss caused by experimental IR injury in Panx1 KO vs. WT mice. We challenged retinas *in vivo* with a 60 minute ischemia followed by reperfusion, which is an established injury model characterized by selective loss of RGCs that occurs within 7 days after reperfusion [41], [42]. A separate group of animals were analyzed 14 days after reperfusion to test whether the Panx1 ablation provides lasting protection, rather than just delaying neuronal loss. RGC densities were assessed across the retina using direct counting of ClassIII β-Tubulin -labeled cells (details in Materials and Methods). RGC densities in corresponding regions of experimental and control retinas were compared and RGC loss rates were calculated for three eccentricity regions of the retina: center, middle and periphery. Protection by the Panx1 ablation was rather even across the retina, with only a slight tendency to a decrease at the periphery (Fig. S4). Therefore, we used averaged RGC loss rates to compare different genotypes. Our data showed that in the WT mice, RGC survival in IR-injured retinas averaged 70.3±0.9% at 7 days after injury. Panx1 knockout strains showed significant increase in neuronal survival 7-days after reperfusion: 98.5±4% (p<0.01) in Panx1 CKO and 95.7±3% (p<0.01) in Panx1 KO (Fig. 2A,B). At 14 days post-reperfusion, RGC survival was also significantly higher in Panx1 KO retinas, averaging 93.4±0.1% vs. 71.6±1.6% (p<0.01) in control WT mice. Thus, the absence of Panx1 protected RGCs from the loss to the IR injury.

We observed no statistically significant difference in RGC survival in CKO and global KO in IR-challenged retinas (Fig. 1B). There was even a small trend toward an increased survival in CKO vs. KO retinas. These findings suggested that Panx1 opening represents an intrinsic mechanism of neuronal death, and did not involve other cell types. To test this hypothesis, we studied survival of pan-purified primary RGCs. RGCs were purified from P7 neonatal mice using the two-step immunopanning technique, as described previously [43], [44] and challenged *in vitro* with transient 4 hour-long oxygen and glucose deprivation (OGD). This model allows for the study of neurons under well controlled conditions that mimic retinal IR injury, including intracellular glucose and ATP depletion, an increase in intercellular Ca^2+^, ER and oxidative stress, followed by cell death by necrosis and apoptosis [45], [46]. In line with our prediction, our data showed significantly higher survival rate in the KO and CKO vs. WT cells (p<0.02, Fig. 2 C). The average RGC survival rate was 46±2% in WT cultures. Survival improved by the average of 22% and 29% in the CKO and KO cells, respectively. It is worth noting that the difference between the two genotypes was not statistically significant, indicating that the deficiency of Panx1 in RGCs is, indeed, responsible for their increased survival. The analysis of dying cells using AnnexinV and propidium iodide (PI) staining revealed that necrotic (PI-positive) cells averaged 16±0.3% in WT RGCs cultures; whereas in the CKO and KO culture, this value has dropped to 4.6±5% and 0.5±1%, respectively. Apoptotic (AnnexinV-positive) cells averaged 40±2% in WT vs. 27.4±3.7% in Panx1 CKO and 24.4±2.3% in Panx1 KO cultures challenged by OGD.

### Panx1 channel permeates RGCs challenged by OGD

Panx1 forms a large non-specific pore, which provides the conduit to ions, dyes and small molecule metabolites with molecular weight up to 1 kDa [16], [47], [48]. In isolated brain neurons, the opening of the Panx1 channel was shown to permeate the plasma membrane in response to 15 minutes of ischemia [8], [20], thus allowing molecules to cross the plasma membrane [35], [36]. Here, we tested whether a membrane-impermeable dye calcein-488AM (MW 660 Da) will leak from RGCs challenged by OGD and if the leakage can be blocked by Panx1 ablation. In the cytoplasm, this dye is de-esterified, becomes hydrophilic and stays trapped inside the cell. Opening of a large pore or channel induces time-dependent exit of the dye from cells, which can be detected by fluorescence microscopy [8], [20].

**Figure 3:**
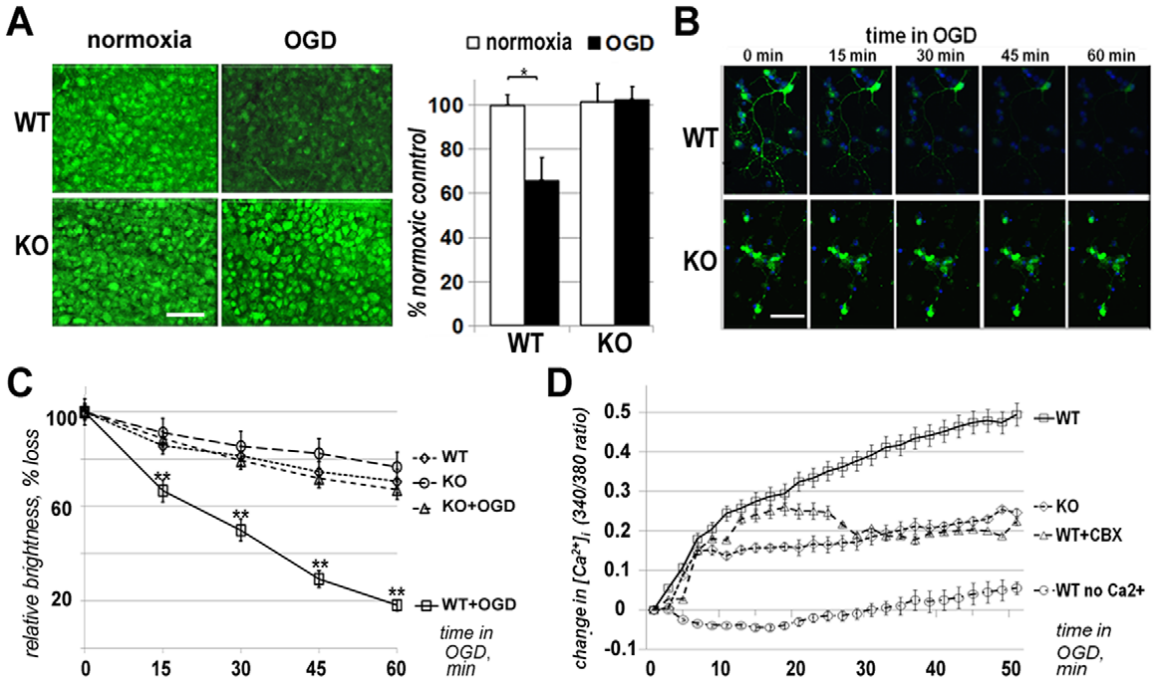
Panx1 KO blocks RGC plasma membrane permeation after ischemia. **A** Representative confocal images of the ganglion cell layer in whole retina flat mounts preloaded with calcein-488. Images were taken after 30 min normoxic or OGD treatment in a hypoxic chamber. Scale bar, 25 µm. Graph shows total calcein fluorescence intensity (mean ± SE, n = 4) after treatment of WT or Panx1 KO retinal explants. The reduction of fluorescence after OGD indicates the dye leakage in WT. **B**. Real-time confocal images of cultured primary RGCs loaded with calcein-488. Shown are representative series of three independent experiments. **C**. The dynamics of calcein-488 dye efflux calculated as the change in fluorescence intensity recorded from at least 50 individual cells in three independent experiments. **D** Intracellular Ca^2+^ changes were measured in cultured primary RGCs loaded with ratiometric fura-2 dye. OGD was induced at the “0” time point by replacing culture media with oxygen-depleted glucose-free media supplemented with 10 mM 2-deoxyglucose and 1 mM KCN. Fluorescence measurements were taken at 510 nm with excitations at 340 and 380 nm in real time with 20 sec intervals. Traces represent the average values (mean ± SE) of the 340/380 ratios obtained from a minimum of 30 individual RGC cells in a minimum of three biological repeats. In WT cells Panx1 blockade was induced by application of 10 µM CBX 20 minutes prior to recording.

We loaded whole retina explants with calcein-488 and measured the leakage after 30 minutes of OGD (de-oxygenized media lacking glucose) in an ischemic chamber. Each pair of WT and Panx1 KO retinas was prepared and challenged by OGD in parallel to avoid technical deviations. After the exposure to OGD, retinal explants were immediately imaged by confocal microscopy. The total fluorescence in the 30 µm-thick optical slice of the inner nuclear layer (INL) was recorded, as detailed in Materials and Methods. We found that OGD caused an average of 33% reduction in total fluorescence in the ganglion cell layer that resulted from dye leakage in WT retinal explants (Fig. 3A). In contrast, retinas obtained from the Panx1 KO mice showed a near-complete blockade of the leakage.

The ganglion cell layer contains a mixed population of RGC, amacrine and glial cells, all of which express different level of pannexins. Therefore, quantitative analysis of the dye leakage from an individual cell type is challenging when performed in retinal explants. To detect whether ganglion cells become permeated, we performed OGD challenge experiments in primary cultures of RGCs *in vitro*. The neurons were pre-loaded with calcein-488AM were exposed to OGD (de-oxygenized media lacking glucose with constant N_2_ bubbling) and dye leakage was monitored by time-lapse confocal microscopy for 60 minutes. Real-time imaging of dye-loaded RGCs revealed a rapid permeation by OGD with a statistically significant drop in calcein-488 fluorescence (leakage) detected as early as 15 min of ischemia (Fig. 3B,C). Using untreated (normoxic) WT RGCs as a reference to normalize for photobleaching, the calculated rate of leakage was 19.2% at 15 minutes, 31.6% and 52.5% at 30 and 60 minutes of OGD, respectively. In contrast, the OGD-challenged Panx1 KO cells showed very slow reduction in fluorescence that closely matched the effect of photobleaching in normoxic cells (Figs. 3C). The calculated changes in calcein-488 fluorescence (2.0% at 30 min and 3.6% at 60 min) were not statistically significant, indicating that the dye remained trapped inside the OGD-treated Panx1 KO cells.

Next, we investigated whether Panx1 ablation influences intracellular [Ca^2+^]_i_ dynamics in ischemia. We used real-time ratiometric fura-2 dye measurements in primary cultured RGCs (Fig. 3D). We found that in WT cells OGD induced a biphasic increase in intracellular free calcium [Ca^2+^]_i_. The first phase occurred within 10 min of OGD, while the second phase was slower and continued for the duration of recording (up to 1 h). Since both phases were not observed when the cells were perfused by Ca^2+^-free medium, we attributed the [Ca^2+^]_i_ increase to the influx of Ca^2+^ across the plasma membrane. In the Panx1 KO cells the first phase was eliminated and the second phase was notably suppressed. A similar effect was observed upon treatment of WT neurons with 10 µM CBX. Thus, Panx1 opening mediates plasma membrane permeability in ischemic RGCs to small molecules such as dyes and ions.

### Panx1 is essential for inflammasome activation in the retina

**Figure 4:**
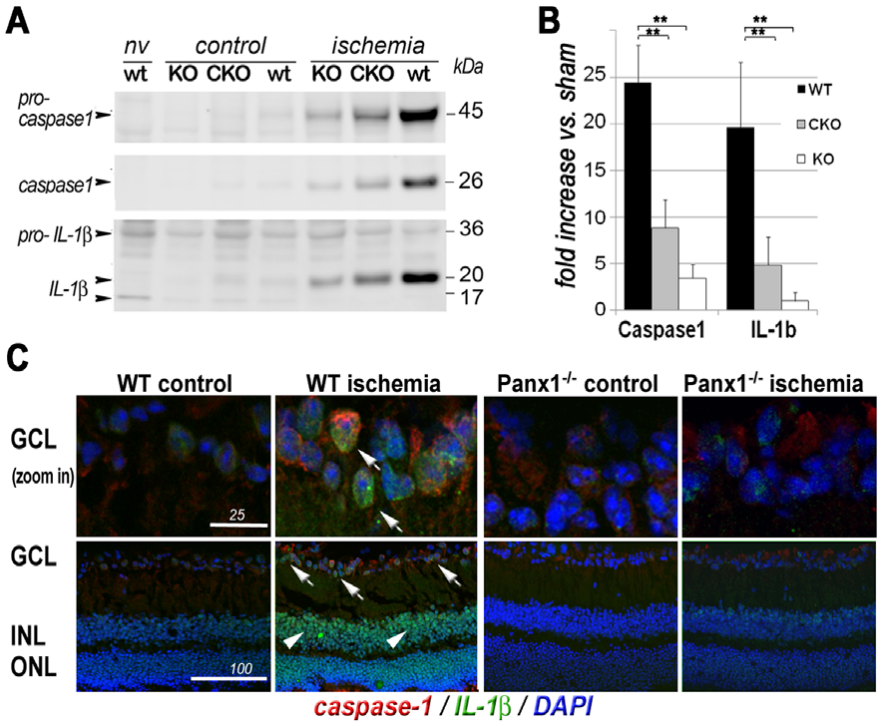
Suppression of IL-1β production and inflammasome activation in Panx1 KO retinas. **A.** The levels of activated caspase-1 and the mature IL-1β proteins observed 3 h after reperfusion in WT, Panx1 KO (KO) and Panx1 CKO (CKO) retinas. **B.** Quantification of the changes in band intensities from western blots in figure A (n = 3, ** p<0.05). **C.** The IHC analysis showing active IL-1β (17 and 20 kDa isoforms, arrowheads) in the cells of the inner retinal layer (INL) after IR challenge. Caspase1-specific (red) and IL-1β-specific (green) staining co-localized with RGCs and other cells in the ganglion cell layer (GCL), IL-1β staining also labeled cells in the INL. DAPI staining for nuclei is blue. Scale bar, 50 µm.

Panx1 has been shown to directly interact and regulate the activation of the inflammasome protein complex in the brain neurons and astrocytes [23], [26], [27]. However, this remains controversial, particularly considering the most recent data published when our manuscript was under review [49]. We examined transcriptional activation of the *Il1b* gene, post-translational activation of interleukin-1β and the key inflammasome protease caspase-1 in response to IR. Retinal lysates from naïve, IR-challenged and sham-operated contralateral eyes of WT and Panx1KO mice were analyzed using quantitative PCR, Western blot and IHC. In addition, we examined the expression of the other inflammasome components, adaptor protein ASC (apoptosis-associated speck-like protein containing a CARD) and NALP1 (NAcht leucine-rich-repeat protein 1) in RGCs. In the WT retinas harvested at 3 hours after reperfusion, we observed: 1) a significant increase in the expression of the *Il1b* gene (Fig. S5), 2) accumulation of pro-caspase-1 and mature caspase-1 (Fig. 4A,B) and 3) accumulation the mature IL-1β. An increase in the levels of both 45 kDa pro-caspase-1 and its 26 kD cleavage product indicated activation of the inflammasome. Consistently, IHC showed a dramatic increase in the caspase-1 and IL-1β labeling, a large proportion of which localized to the cells in the ganglion cell layer (Fig. 4C). Furthermore, IHC co-localization analysis performed in primary cultures confirmed that RGCs, indeed, expressed high levels of caspase-1 and IL-1β after OGD challenge (Fig. S6). Lower levels of IL-1β immunoreactivity were also found in other retinal layers, a pattern consistent with the distribution of secreted proteins (Fig. 4C). The expression of ASC and NALP1 and their co-localization with RGC-specific marker ClassIII β-Tubulin was also detected by IHC (Fig. S7). However, no significant induction of the ASC and NALP1 proteins was detected after IR (data not shown). These observations indicate that robust activation of inflammasome and IL-1β production is a part of the acute response of the retina to the IR injury.

We then compared retinal lysates from WT and Panx1KO mice to test if Panx1 is involved in the regulation of inflammasome activation in IR. We found that activation of all components of the inflammasome complex was suppressed in the Panx1 KO retinas and the inhibition was stronger in global Panx1 KO vs. Panx1 CKO animals. Sham-operated fellow eyes showed no statistically significant changes in inflammasome protein accumulation and processing. Thus, our results indicate that Panx1 is essential for inflammasome activation in the IR-challenged inner retina, particularly in RGCs.

## Discussion

In this study, we investigated the role of the Panx1 channel in the pathophysiology of global retinal ischemia followed by reperfusion. We have demonstrated that RGCs in Panx1-deficient animals are remarkably resistant to ischemia, providing strong evidence that the Panx1channel is an essential player in pathophysiology of IR injury in neurons. We also identified two distinct neurotoxic mechanisms that are mediated by Panx1: RGC membrane permeation to small molecules and ions (Fig. 3) and activation of the inflammasome (Fig. 4).

Our data also show that the mechanisms of RGC injury in IR are intrinsic to these neurons and are caused by the opening of the endogenous Panx1 channels. This notion is supported by three lines of evidence. First, neuronal Panx1 knockout provided the same, if not higher, degree of *in vivo* protection as the global Panx1 ablation (Fig. 2B). Second, primary Panx1-deficient RGCs possessed increased survival, decreased rates of apoptosis and near complete suppression of necrosis after exposure to OGD *in vitro* (Fig. 2C). Finally, the IHC data show that RGCs, which are the most vulnerable to ischemia among retinal neurons, possess the highest levels of Panx1 expression in the retina (Fig. 1, Fig. S2). The latter is consistent with our earlier report that utilized *in situ* hybridization and gene expression analysis in purified primary RGCs [50].

### The role of Panx1 in rapid membrane permeation induced by ischemia

The role of Panx1 channel opening in the ischemia-induced membrane permeation was first demonstrated using erythrocytes and isolated hippocampal neurons [8], [20], [51]. In those experiments Panx1 opening occurred within the first 15 min of ischemia. However, the ability of Panx1 to permeate neurons *in vivo* remained controversial after Madri and co-authors [52] showed that in hippocampal slices Panx1-dependent permeation was significantly slower (90 vs. 15 min) than in isolated neurons. Our experiments showed that the average rate of dye leakage from the ganglion cell layer in the retinal wholemounts was significantly suppressed in Panx1 KO retinas (Fig. 3A). Similar experiments performed in real time in cultured primary RGCs showed that 15 minutes of ischemia (OGD) was sufficient for the induction of a robust Panx1 channel opening; 30 min of ischemia averaged 33% and 31.6% of total calcein 488 fluorescence reduction *ex vivo* and *in vitro*, respectively.

As expected, the Panx1-mediated permeation of plasma membrane in oxygen- and glucose-deprived RGCs also altered ionic homeostasis and increased the rate of [Ca^2+^]_i_ accumulation. Our data showed that the difference between the WT and Panx1 KO RGCs became statistically significant only after 10 minutes of OGD; only the second phase of Ca^2+^ accumulation was blocked by application of 10 µM CBX or by Panx1 ablation (Figs. 3D). Thus, our findings are consistent with the model where Panx1 channel-mediated permeation of the plasma membrane is rapid [8], [20], [51].

### Panx1 mediates neuronal injury in the retinal IR model

We demonstrated that Panx1 in RGCs contributes to pathophysiology of acute retinal ischemia. The IR injury in the retina is known to cause a delayed neuronal cell loss, which occurs several days after primary insult, while secondary degeneration extends for weeks [41], [53], [54]. Up to seven days post-reperfusion the loss is specific to RGCs, while amacrine cells and other neurons succumb later [41]. Our results showed near-full protection of RGCs one week after reperfusion in both global and conditional Panx1 deletion. Furthermore, RGCs remained protected for as long as 14 days (Fig. 2B), showing that RGC loss is not merely delayed but actually prevented by Panx1 ablation. These results are consistent with the reports of neuroprotection by global pharmacological blockade of hemichannels in brain ischemia and inflammation models [9], [13], [54], [55]. Thus, Panx1 plays a significant role in ischemic pathology of the retina, which evidently differs from ischemic stroke in the brain, as reported recently [49].

Long-term protection implies that in addition to initial acute injury mediated by rapid Panx1 channel opening, Panx1mediates a long-acting mechanism(s) of neurotoxicity. One possibility is that Panx1 activation triggers neurotoxic signaling that facilitates secondary degeneration. Indeed, several toxicity pathways, such as [Ca^2+^]_i_ accumulation, oxidative ROS production [54], excitotoxicity [56], necrotic cell signaling [57], TLR-4 signaling [58] and pro-inflammatory cytokine induction [28], [59] were characterized in post-IR retina. Interestingly, OGD induced necrosis in the WT RGC cultures but not in Panx1-deficient neurons (Fig. 2 C). The lack of necrotic cells in post-ischemic retinas indicates inhibition of necrotic cell signaling, which is a novel degeneration pathway recently characterized in retinal ischemia [57], [60] and cone-rod dystrophy [61]. Since necrotic death in the ischemic retina is caused primarily by ATP-depletion [60], we speculate that the protective effect of Panx1 deletion likely involves preservation of intracellular ATP levels.

### Panx1 is essential for activation of neuronal inflammasome after IR injury

Our present work shows that the neuronal inflammasome is activated by retinal IR, as detected by two major markers of inflammasome activation: caspase-1 proteolysis and production of the mature IL-1β. We detected a change in the levels of precursor and mature caspase-1, which signifies the activation of the inflammasome complex, and the timing of this activation coincided with the increased expression and processing of IL-1β. Additional evidence is the expression of inflammasome proteins ASC and NALP1, detected in RGCs by immunohistochemistry. Mature IL-1β is released and accumulated in the retina, and the IHC data show that RGCs are a major inner retina cell type producing IL-1β. As confirmed by Western blot and co-localization analysis in the IHC data (Fig. S6), RGCs also show an increased expression of caspase-1 in response to retinal IR injury (Fig. 4C). Our findings are consistent by previous reports that observed IL-1β production [28], [43] and increased expression of caspase-1 in the inner retina of post-ischemic rodent eyes [62]. The major markers of inflammasome activation were considerably suppressed in the Panx1 KO retinas, indicating that Panx1 is required for activation of this complex.

Panx1 was previously shown to co-immunoprecipitate with components of NALP1-containing inflammasome [63] and to be involved in regulation of its activity [35], [36]. Inflammasome was shown to be activated by diverse factors. For example, in injured tissues, it is activated by danger-associated molecular patterns (DAMPs), the stress-induced molecules that are released from dying cells and bind to pattern recognition receptors (PRRs) [64], [65]. Significantly, inflammasome activation in brain astrocytes and neurons is implicated in the pathology of brain trauma and thromboembolic stroke [26], [27], [66]. However, in other cell types such as in monocytes, the active role of Panx1 in regulating inflammasome activity is currently under debate [22], [49], [67].

Production of mature interleukins requires activation of gene expression and subsequent processing of the precursor proteins by caspase-1. Several lines of experimental evidence indicated that Panx1 is involved in both these processes. First, transcriptional activation of interleukin precursors via MyD88/NF-kappaB pathway required stimulation of PRRs, for example the NOD-like receptors, which are intracellular and require cytosolic delivery of extracellular DAMPs [66], [68]. According to Kanneganti and co-authors [35], such a delivery occurs through the Panx1 channel. Second, activation of caspase-1 was shown to require the direct interaction between Panx1 and components of inflammasome in brain cells [23], [63]. The model in which Panx1 contributes to both transcriptional and post-translational activation of IL-1β by inflammasome, is consistent with our results (Fig. 4) showing that Panx1 is essential for IL-1β processing and production in the retina. Interestingly, it was previously reported that blockade of caspase-1 by intravitreal injection of the selective peptide inhibitor provided a similar level of neuroprotection against retinal IR as Panx1 ablation in our experiments [69]. Taken together with previously published findings, our study of inflammasome in WT and Panx1 knockout mice show that 1) inflammasome activation is a novel neurotoxicity pathway in retinal IR and 2) inflammasome activation is facilitated by Panx1.

In conclusion, our results show that Panx-1 mediates neuronal IR injury through a mechanism that involves acute permeation of plasma membrane and activation of inflammasome. Our findings demonstrate that this pathway is intrinsic for RGCs. Membrane permeation via Panx1 contributes to acute injury by mediating ionic and metabolic disbalance and triggers long-term toxicity mechanisms such as cytokine production by the neuronal inflammasome. Panx1 ablation effectively suppresses inflammasome and IL-1β production *in vivo* in post-ischemic RGCs, which correlates with neuroprotection.

## Materials and Methods

### Animals

All experiments and post-surgical care were performed in compliance with the NIH Guide for the Care and Use of Laboratory Animals and according to the University of Miami IACUC approved protocol. Wild type (WT) animals used in our experiments were 2–3 months old male mice of C57BL/6 background; 6 animals per group). Panx1^fl/fl^ mouse line with three LoxP consensus sequences integrated into Panx1 gene was generated in collaboration with the transgenic facility of the National Institute of Child Health and Development (Bethesda, MD), using our own recombinant DNA constructs. Knockout mice with global (CMV-Cre/Panx1) and neuron-specific conditional (Thy1-Cre/Panx1) inactivation of Panx1 were bread in the University of Miami facility. Retinal tissues for immunopanning were obtained from neonatal P5–P7 pups. “Floxed” mouse lines were generated at the NIH NICHD Transgenic Mouse Core Facility and transferred to the UM DVR for further breeding with Cre-expressing lines. These mice were back-crossed to C57Bl6 background for at least 5 generations prior to experiments. Mice were housed under standard conditions of temperature and humidity, with a 12-hour light/dark cycle and free access to food and water.

### Isolation of primary RGCs

P5–7 old pups were euthanized according to the University of Miami IACUC approved protocol, eyes were enucleated and retinas were mechanically dissected out. RGCs were isolated according to the two-step immunopanning method. Briefly, the whole retinas were incubated in papain solution (16.5 U/ml) for 30 min. In the next step macrophage and endothelial cells were removed from the cell suspension by panning with the anti-macrophage antiserum (Axell Accurate Chemical Corp., Westbury, NY). RGCs were specifically bound to the panning plates containing anti-Thy1.2 antibody, and unbound retinal cells were removed by washing with DPBS. Purified RGCs were released by trypsin incubation and grown in Neurobasal/B27 media (Invitrogen, USA).

### Transient retinal ischemia-reperfusion (IR) model

After anesthesia with intraperitoneal ketamine (80 mg/kg) and xylazine (16 mg/kg), pupils were dilated with 1% tropicamide–2.5% phenylephrine hydrochloride (NutraMax Products, Inc., Gloucester, MA), and corneal analgesia was achieved with 1 drop of 0.5% proparacaine HCl (Bausch & Lomb Pharmaceuticals, USA). Retinal ischemia was induced by increasing intraocular pressure (IOP) above cystolic blood pressure (to 120 mm Hg) for 60 minutes. IOP was elevated by direct cannulation of the anterior chamber of the eye a 33-gauge needle attached to a normal (0.9% NaCl) saline-filled reservoir raised above the animal. The contralateral eye was cannulated and maintained at normal IOP to serve as a normotensive control. Complete retinal ischemia, evidenced by a whitening of the anterior segment of the eye and blanching of the retinal arteries, was verified by microscopic examination. After needle removal, erythromycin ophthalmic ointment (Fougera & Atlanta, Inc., Melville, NY) was applied to the conjunctival sac. Mice were sacrificed in 7 or 14 days after reperfusion by CO_2_ inhalation under anesthesia.

### Oxygen/glucose deprivation (OGD) model

After replacement of the media with fresh glucose, amino acids, vitamins and sodium pyruvate-free Neurobasal media with B27 supplements (Invitrogen, USA), RGCs were exposed to hypoxia by replacing of the Neurobasal media with glucose-free OGD media (Hanks' balanced salt solution with sucrose substituting glucose, deoxygenated by 1 h bubbling with nitrogen). Cells were placed into hypoxic chamber (BioSpherix) for 4 h at 37°C, after which the culture medium is then changed for fresh Neurobasal/B27 media, 4 or 24 h incubation in a 5% CO2 atmosphere at 37°C (“reperfusion” phase). For real-time imaging in RGC cultures, cover slips with attached cells were placed into class-bottom microscopy chambers (Lab-Tek II Chamber Slide). To achieve OGD conditions in these chambers, normoxic media was substituted with the deoxygenized glucose-free media, oxygen was removed by continuous nitrogen bubbling through a circular perforated microtube line glued to the bottom. Each chamber has been calibrated and tested by direct O_2_ measurements with OxyLab pO2 oxygen sensor (Oxford Optronix Ltd.) to achieve pO_2_<5 mmHg (time “0” for real-time recordings) 10 minutes after the media change (see Fig. S8 for the pO_2_ kinetics in the chamber with nitrogen bubbling).

### Dye transfer tests

Media, cells or tissues were loaded with membrane-impermeable Calcein 488 AM fluorescent dye (491 nm excitation/509 nm emission). RGCs were plated at a density of 8×10^4^ cells/well on 24-well plates with 12 mm glass coverslips pre-coated with poly-L-lysine (10 µg/mL) and incubated in Neurobasal/B27 media overnight. Retinas were dissected into Neurobasal/B27 media, flat-mounted on the glasses bottoms pre-coated with Cell-Tek Cell and Tissue Adhesive (BD Biosciences) and stored in oxygenated chambers at 32°C prior to labeling. For dye loading, cells and whole retinas were incubated with 5 µM calcein-AM488 in artificial cerebrospinal fluid (ACSF, contains (in mM) 150 NaCl, 10 glucose, 5 KCl, 2 CaCl2, 1 MgCl2, 10 HEPES at pH 7.4) for 30 min at 37°C in humidified incubator under 5% CO2. Cells were washed with ACSF solution three times to remove the extracellular calcein-AM. The coverslips with calcein-loaded cells were removed and transferred into glass-bottom hypoxia chamber for OGD challenge and the fluorescence imaging analysis. After loading, the chambers with retina wholemounts were placed into hypoxic chamber (BioSpherix) for 30 minutes at 37°C in OGD solution. Cells and retinas were imaged at spectroscopic Leica TCP AOBS SP5 confocal microscope (Leica Microsystems, Exton, PA). All the images from each experiment were captured under identical microscope settings on the same day and further analyzed using Leica image analysis software. The fluorescence intensity values from 50 individual cells in each experiment (n = 3) were determined by real-time recording using Leica proprietary image analysis software, the mean values and standard error (SE) were calculated. Single cell calcein dye leakage dynamics were recorded using 10 minutes after media change and the beginning of N_2_ bubbling as t = 0. The OGD solution, containing (in mM) 150 NaCl, 10 Sucrose, 5 KCl, 2 CaCl2, 1 MgCl2, 10 HEPES at pH 7.4, was de-oxygenated by N_2_ bubbling for at least 1 hr before experiments. To normalize for the effects of photobleaching, the recorded intensity values were compared to the signal decline in the control baseline curve, recorded from similarly processed but untreated (normoxic) cells, where the changes in fluorescence intensity were due to photobleaching. Individual retinas were sampled randomly to collect a total of 20 images located at the same eccentricity in the four retinal quadrants, using a 20× objective lens. The fluorescence intensity values from 20 images in each experiment (n = 3) were determined using the Leica software.

### Calcium imaging

Calcium imaging was performed on acutely isolated neonatal (P7) retinal ganglion cells, as described previously [70]. One-day old cultures on laminin-coated coverslips were incubated in Neurobasal media containing 1 µM fura-2AM for 60 minutes at 37 degrees C in the dark. This was followed by a 30 min wash in dye-free ACSF media to permit de-esterification of fura-2AM. The cover slips were then secured in a flow chamber and mounted on the stage of a Nikon TE2000 inverted fluorescence microscope. The cells were perfused with ACSF media and subjected to OGD treatments as required by the experiment. Images were collected using 20× UV objective lens in real time every twenty seconds for 60 minutes. The excitation wavelengths were 340 and 380 nm provided by a 150 W Xenon arc lamp (DG4, Sutter Instruments) and the emission was set at 510 nm. Free Ca^2+^ concentration was determined from the fluorescence measurements using the fura-2 Ca^2+^ imaging calibration kit (Molecular Probes) according to manufacturer's instructions. Data acquisition and F340/F380 ratio calculations were performed using MetaFluor software using regions of interest (ROIs) encompassing individual RGCs at 3×3 binning. Background fluorescence was measured in similarly sized ROIs in neighboring areas devoid of cells and subtracted from ROI readings. OGD conditions for real time imaging were achieved by replacing with oxygen-free, glucose-free ACSF media (equivalent amount of sucrose was added to maintain osmolarity) and constant bubbling of media and chamber with N_2_. In addition, to ensure full blockade of glycolysis in shallow perfusion chamber, 10 mM 2-deoxyglucose and 1 mM KCN were added as described earlier [71]. Glutamate (100 µM) was added at the end of each individual experiment to identify responsive live cells; the addition of 10 mM 2-deoxyglucose and 1 mM KCN in full ACSF media not induce any responses in RGCs (data not shown).

### Antibodies

Three different species of antibodies raised against the C-terminal portion of Panx1 were used in this study: affinity purified rabbit anti-Panx1 CT-395(Px-34) antibodies [38] provided by Dr. D.W. Liard (University of Western Ontario, Canada) and rabbit polyclonal anti-human Pannexin1 antibodies purchased from Chemicon, Inc and AbnOvation, Inc. We used 1∶1000 dilution for immunohistochemistry (IHC) and 1∶5000 for western blot. The rabbit polyclonal antibodies against ASC and NALP1 proteins were provided by Dr. Rivero Vaccari. The following commercially available antibodies were used: anti-caspase-1 (Abcam), anti-IL-1β (Cell Signaling Technology), anti-XIAP (BD Transduction Labs); anti-GFAP (Sigma), and anti-CD11b (Millipore), anti-Neuronal Class III β-Tubulin (Covance). Secondary species-specific fluorescence AlexaFluor dye-labeled antibodies for confocal were purchased at Invitrogen/Molecular Probes, USA.

### RGCs loss

The analysis of the RGCs loss in the inner retina was performed using a specific anti-ClassIII β-Tubulin staining. Whole retinas were flat-mounted, coverslipped and specific fluorescence in the inner retina was imaged. To avoid topological irregularities, stacks of 5 serial images collected for depth 0–30 µm were collapsed to generate the “maximum projections” (standard feature of the Leica LAS AF software), where all imaged cells appear in sharp focus. These images were used for RGC counts with MetaMorph (Universal Imaging Co., USA) software, after image thresholding and manual exclusion of artifacts. Individual retinas were sampled randomly at 20 random fields in three regions/four retinal quadrants at the same eccentricities (5 at 0.5 mm, 10 at 1.0 mm and 5 at 1.5 mm from the optic disk) using 20× objective lens. RGC loss was calculated as percentages of β-Tubulin -positive cells in experimental eyes relative to sham-operated contralateral control eyes that was cannulated but maintained at normal IOP. The data from five animals were averaged for each group and genotype.

### Neuronal death assay

The percentages of necrotic and apoptotic cells after OGD challenge were determined using the Vybrant Apoptosis Assay Kit #2 (Invitrogen, USA). Cells were imaged using a Leica TCP SP5 confocal microscope and counted using Metamorph imaging software. The percentage of necrotic cells (Annexin V and PI) and apoptotic cells (only Annexin V) relative to the total number of cells was determined for each of ten images.

### Supplement Methods

We used standard methods for quantitative RT-PCR, Western blot, immunohistochemistry and statistical analyses (see Methods S1). The sequences of PCR primers are provided in Table S2.

## Supporting Information

Table S1 **RGC density in retinas of different genotypes.**
(DOC)

Table S2 **PCR primers utilized in this study.**
(DOCX)

Methods S1 (DOC)

Figure S1 **Panx1 conditional knockout construct.**
(PDF)

Figure S2 **Immunohistochemical detection of the Panx1 protein.**
(PDF)

Figure S3 **Physiological (ERG) tests of retinal function.**
(PDF)

Figure S4 **Neuroprotection in different retinal regions of Panx1-deficient animals.**
(PDF)

Figure S5 **Differential activation of the** ***Il1b*** **gene in WT vs. Panx1 KO retinal in response to IR.**
(PDF)

Figure S6 **Co-localization analysis of IL-1β and caspase-1 in primary RGC after OGD.**
(PDF)

Figure S7 **Co-localization analysis of inflammasome marker proteins ASC and NALP1.**
(PDF)

Figure S8 **OGD chamber test for the pO_2_ kinetics.**
(PDF)
