# Supplementary material for: Genetic Ablation of Pannexin1 Protects Retinal Neurons from Ischemic Injury
Source: PLoS One. 2012 Feb 23;7(2):e31991. doi: 10.1371/journal.pone.0031991 (PMC3285635; doi:10.1371/journal.pone.0031991)
Supplement: Table S1 — RGC density in retinas of different genotypes. (DOC) [file pone.0031991.s001.doc]

**Supplement Table S1.** RGC density in retinas of different genotypes

| Genotype | RGC density per field* | SDEV | N |
| --- | --- | --- | --- |
| **WT** | 239.2 | 36.0 | 5 |
| **Thy1-Cre/Panx1** | 225.5 | 24.7 | 5 |
| **CMV-Cre/Panx1** | 239.2 | 20.8 | 6 |

*averaged from 20 fields per retina, at three eccentricities
