# Supplementary material for: Genetic Ablation of Pannexin1 Protects Retinal Neurons from Ischemic Injury
Source: PLoS One. 2012 Feb 23;7(2):e31991. doi: 10.1371/journal.pone.0031991 (PMC3285635; doi:10.1371/journal.pone.0031991)
Supplement: Table S2 — PCR primers utilized in this study. (DOCX) [file pone.0031991.s002.docx]

**Supplement Table S2**. PCR primers utilized in this study

| **Gene** | **Oligonucleotides** | |
| --- | --- | --- |
| ***Il1b*** | Forward | gaccttccaggatgaggaca |
|  | Reverse | aggccacaggtattttgtcg |
| ***Panx1*** | Forward | caagggagaggaccagggc |
|  | Reverse | atctattcttctatgacgctg |
| ***Panx2*** | Forward | gagaaaaagcatacccgccac |
|  | Reverse | gggtgagcagacatggaatg |
| ***Cx50*** | Forward | gcaagagagaaagacagcac |
|  | Reverse | ccagaggcggatgtgtgag |
| ***Actb*** | Forward | CACCCTGTGCTGCTCACC |
|  | Reverse | GCACGATTTCCCTCTCAG |
