# Supplementary material for: Genetic Ablation of Pannexin1 Protects Retinal Neurons from Ischemic Injury
Source: PLoS One. 2012 Feb 23;7(2):e31991. doi: 10.1371/journal.pone.0031991 (PMC3285635; doi:10.1371/journal.pone.0031991)
Supplement: Methods S1 — (DOC) [file pone.0031991.s003.doc]

**Supplement Methods S1**.

*Real-time PCR analysis.* Changes in gene expression were assessed at 3 hours post-reperfusion by real-time PCR analysis, using gene-specific primer pairs (listed in Supplement Table S2), as described previously [65]. Total RNA was extracted using Absolutely RNA Nanoprep kit (Stratagene, USA), reverse transcribed with the Reverse Transcription System (Promega , USA) to synthesize cDNA. Real-time PCR was performed in the Rotor-Gene 6000 Cycler (Corbett Research, Australia) using the SYBR GREEN PCR MasterMix (Qiagen, USA). For each gene, relative expression was calculated by comparison with a standard curve, following normalization to the β-actin (Actb) gene expression.

*Western blot.* Retinas were dissected out, snap frozen in liquid nitrogen and stored at -80ºC. Tissue was homogenized in T-PER buffer lysis buffer (Tissue Protein Extraction Reagent by Thermo Scientific, Inc.) supplemented with complete protease inhibitor (Roche) and tissue debris was removed by centrifugation. Protein concentration was assessed using the BCA kit (Pierce). Equal amount of total protein from each sample was resolved on SDS-PAGE gradient 4-12% Bis-Tris gels and transferred to PVDF membrane (Invitrogen). Blots were blocked in 5% milk in Tris-buffered saline (TBS, pH 7.6), probed with the primary antibody against Panx1 overnight, washed in 0.15% Tween20 in TBS, and incubated for 1h with secondary antibody (1:1,000, Amersham Biosciences, NJ, USA) diluted in TBS. Anti-actin antibodies were used to control the loading. Proteins were visualized using SuperSignal chemiluminescent substrates (Pierce) and quantified using the FUJIFILM software. Data were normalized to β-actin/GAPDH and expressed as a percentage of naïve control.

*Immunohistochemistry*

Eyes were enucleated, incised at the *ora serrata*, fixed in 4% paraformaldehyde for 2 hours and cryoprotected with 20% sucrose. Retinas were sectioned to a thickness of 80 μm on a vibratome (Vibratome, St. Louis, MO) and immunostained protocol as described previously [41,42]. Sections were incubated with a given primary antibody (listed in Supplement Table 1) for 4-16 h, followed by species-specific secondary fluorescent antibodies (AlexaFluor, Invitrogen, USA). Control sections were incubated without primary antibodies. Sections were examined by cofocal microcopy (Leica TSL AOBS SP5 confocal microscope, Leica Microsystems).

*ERG recording.* Scotopic electroretinogram (ERG) was recorded from dark-adapted animals using a UTAS system (LKC Technologies, Gaithersburg, MD). Animals were anesthetized with ketamine/ xylazine (80/16 mg/kg). Pupils are dilated with atropine/phenylephrine HCl (0.1%). During the recording, animals were maintained at 35°C on a heated plate. A contact lens with platinum wire electrode was placed on the cornea, a differential electrode was placed under the skin between eyes, and a ground wire electrode was attached under the skin close to the tail base. Both eyes were recorded simultaneously. Scotopic ERG responses were elicited by 1 ms white flashes of -6.9 and 0.6 log cd-s/m2 log cd**·**s/m^2^ generated by white LEDs in the Ganzfeld sphere of the UTAS system. Inter-stimulus intervals were 30 s.

Photopic ERG responses were recorded after the scotopic ones using same instrument and flash intensities between -0.9 and +0.6 log cd-s/m2. Waveforms were averaged from 5 or more animals of the same genotype, gender and age and used for wave amplitudes and oscillatory potentials.

*Statistical analysis.* Statistical analysis of real time PCR and cell density data was performed with one-way ANOVA followed by Tukey test for multiple comparisons. In case of single comparisons, the Student’s T-test was applied. P values equal to or less than 0.05 were considered statistically significant.
