# Supplementary material for: Genetic Ablation of Pannexin1 Protects Retinal Neurons from Ischemic Injury
Source: PLoS One. 2012 Feb 23;7(2):e31991. doi: 10.1371/journal.pone.0031991 (PMC3285635; doi:10.1371/journal.pone.0031991)
Supplement: Figure S1 — Panx1 conditional knockout construct. (PDF) [file pone.0031991.s004.pdf]

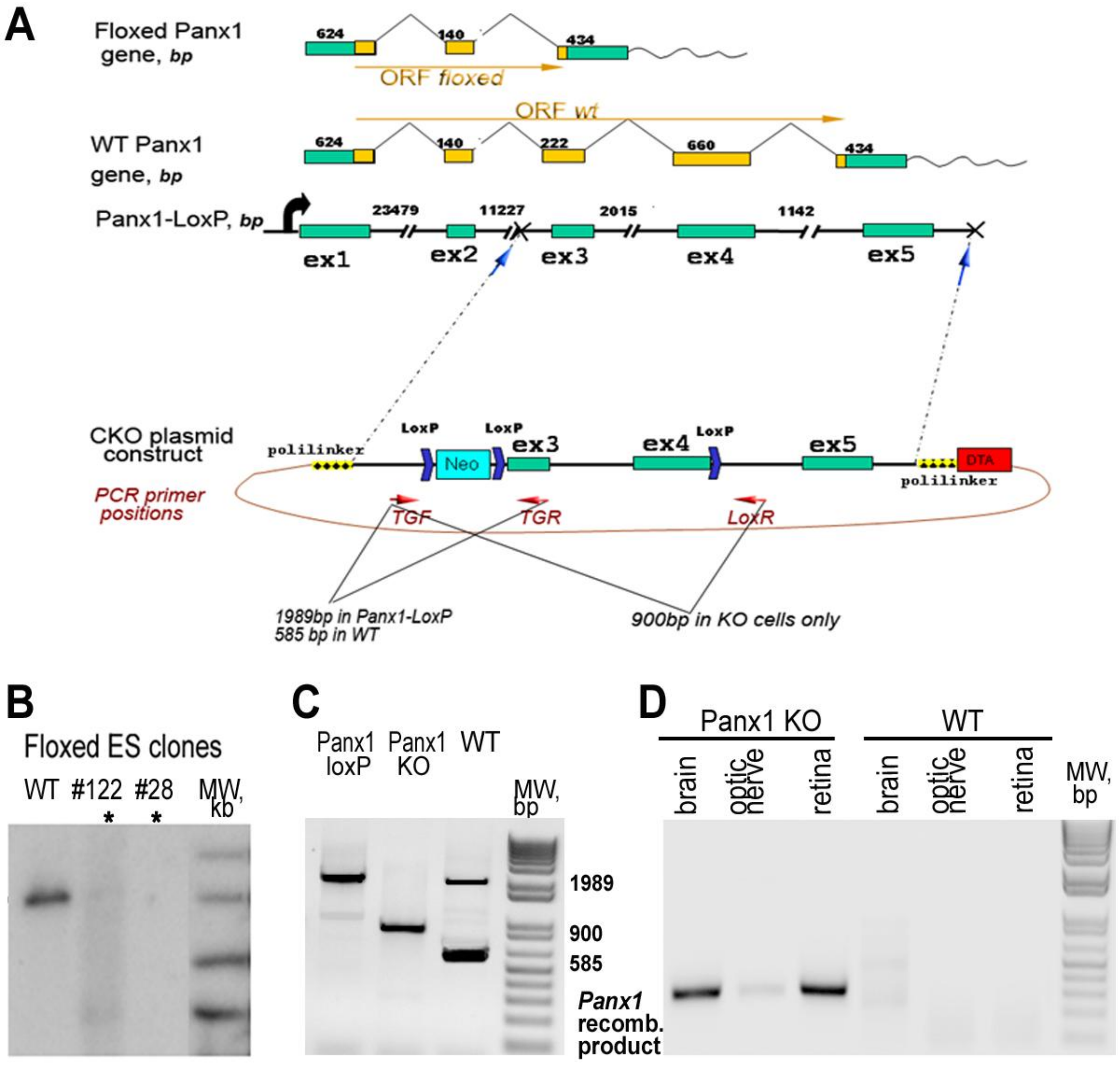

Supplement Figure S1. ***Panx1 conditional knockout construct***. ***A***. *The knockout construct design. The fragment containing Exons 3-5 was flanked by LoxP sites, the Neo resistance selection marker at the left shoulder and DTA "suicide" gene, was inserted into C129 ES cells by homologous recombination. Cre-recombination of the recombinant Panx1 gene eliminates the entire LoxP flanked region.* ***B***. *Southern Blot analysis of floxed ES clones for the Panx1 transcript removal.* ***C.*** *PCR-genotyping Panx1/LoxP and CMV-Cre/Panx1 mice (Panx1 KO).* ***D***. *PCR detection of recombined functionally deficient floxed Panx1 transcript in the tissues of CMV-cre/Panx1 (Panx1 KO) and WT mice, using a set of 2 primers.*
