# Supplementary material for: Genetic Ablation of Pannexin1 Protects Retinal Neurons from Ischemic Injury
Source: PLoS One. 2012 Feb 23;7(2):e31991. doi: 10.1371/journal.pone.0031991 (PMC3285635; doi:10.1371/journal.pone.0031991)
Supplement: Figure S2 — Immunohistochemical detection of the Panx1 protein. (PDF) [file pone.0031991.s005.pdf]

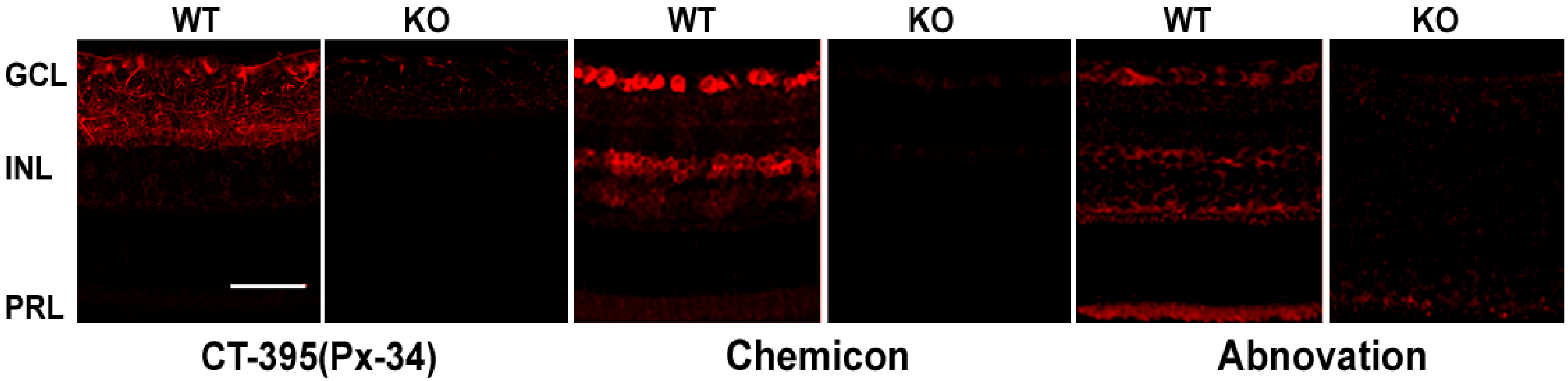

Supplement Figure S2. Immunohistochemical detection of the Panx1 protein *(red) in wild type (WT) and Panx1 KO (KO) retinal slices using different C-terminal antibodies. All three antibodies showed brightest labeling in the ganglion cell layer (GCL) of WT retinas and near-complete lack of labeling in the retinas of Panx1 KO animals. Minor labeling is also present in inner nuclear (INL) and photoreceptor (PRL) layers. The CT-395 antibodies showed some residual immunostaining in Panx1 KO retinal tissues that localized to RGC dendrites. Scale bar, 50 μm*
