# Supplementary material for: Genetic Ablation of Pannexin1 Protects Retinal Neurons from Ischemic Injury
Source: PLoS One. 2012 Feb 23;7(2):e31991. doi: 10.1371/journal.pone.0031991 (PMC3285635; doi:10.1371/journal.pone.0031991)
Supplement: Figure S3 — Physiological (ERG) tests of retinal function. (PDF) [file pone.0031991.s006.pdf]

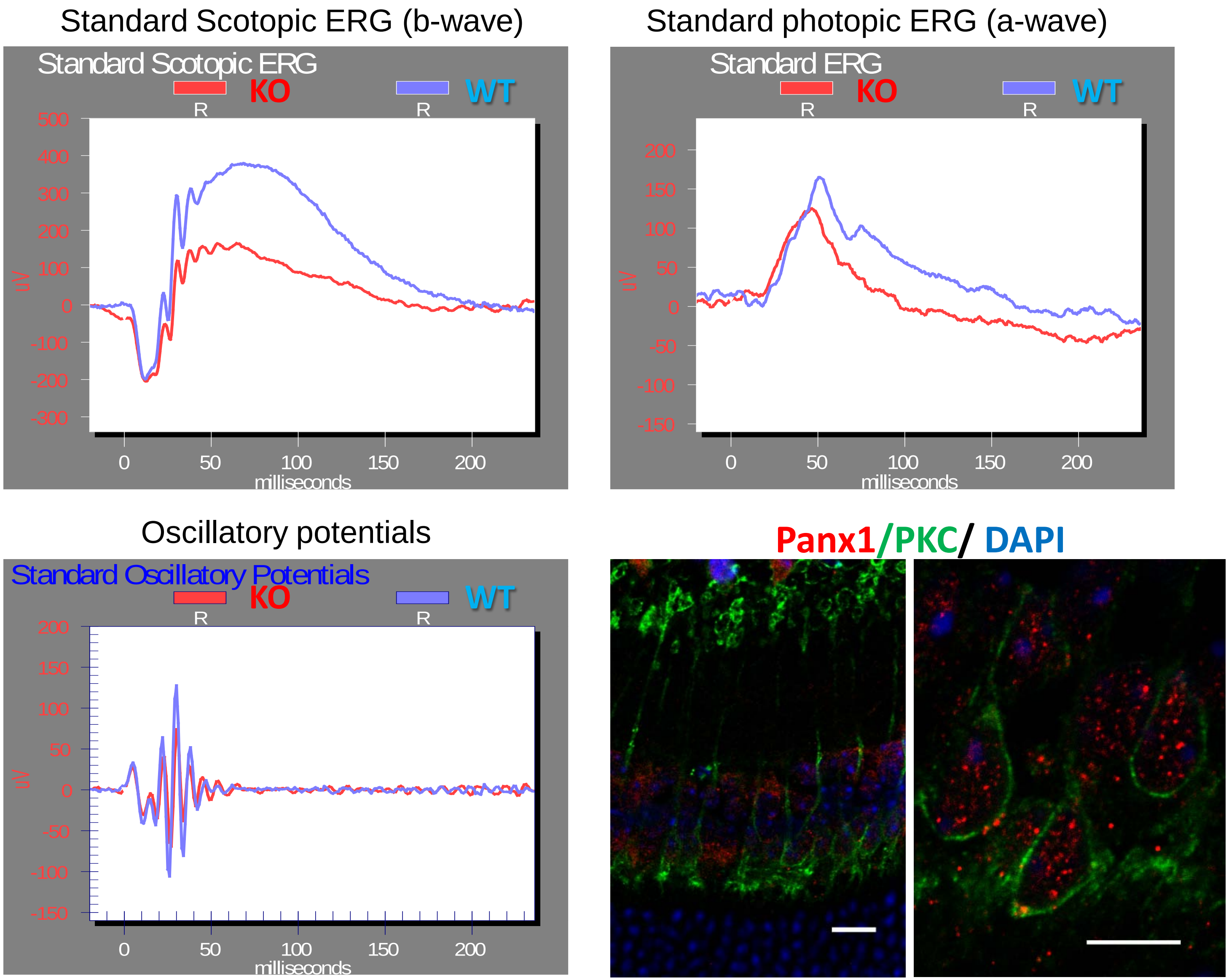

Supplement Figure S3. A-C Physiological (ERG) tests of retinal function. *ERG waveforms for b-wave (A), a-wave (B) and oscillatory potentials averaged for WT (n=6) and Panx1 KO (n=8) animals. Animals were dark-adapted overnight for b-wave recordings.* ***D****. Double Immunohistochemical labeling of the Panx1 protein (red) and bipolar cells (PKC, green) in wild type (WT) retinal slices using antibodies against C-terminal part of Panx1; DAPI staining for nuclei, blue; scale bar, 25 μm*
