# Supplementary material for: Genetic Ablation of Pannexin1 Protects Retinal Neurons from Ischemic Injury
Source: PLoS One. 2012 Feb 23;7(2):e31991. doi: 10.1371/journal.pone.0031991 (PMC3285635; doi:10.1371/journal.pone.0031991)
Supplement: Figure S4 — Neuroprotection in different retinal regions of Panx1-deficient animals. (PDF) [file pone.0031991.s007.pdf]

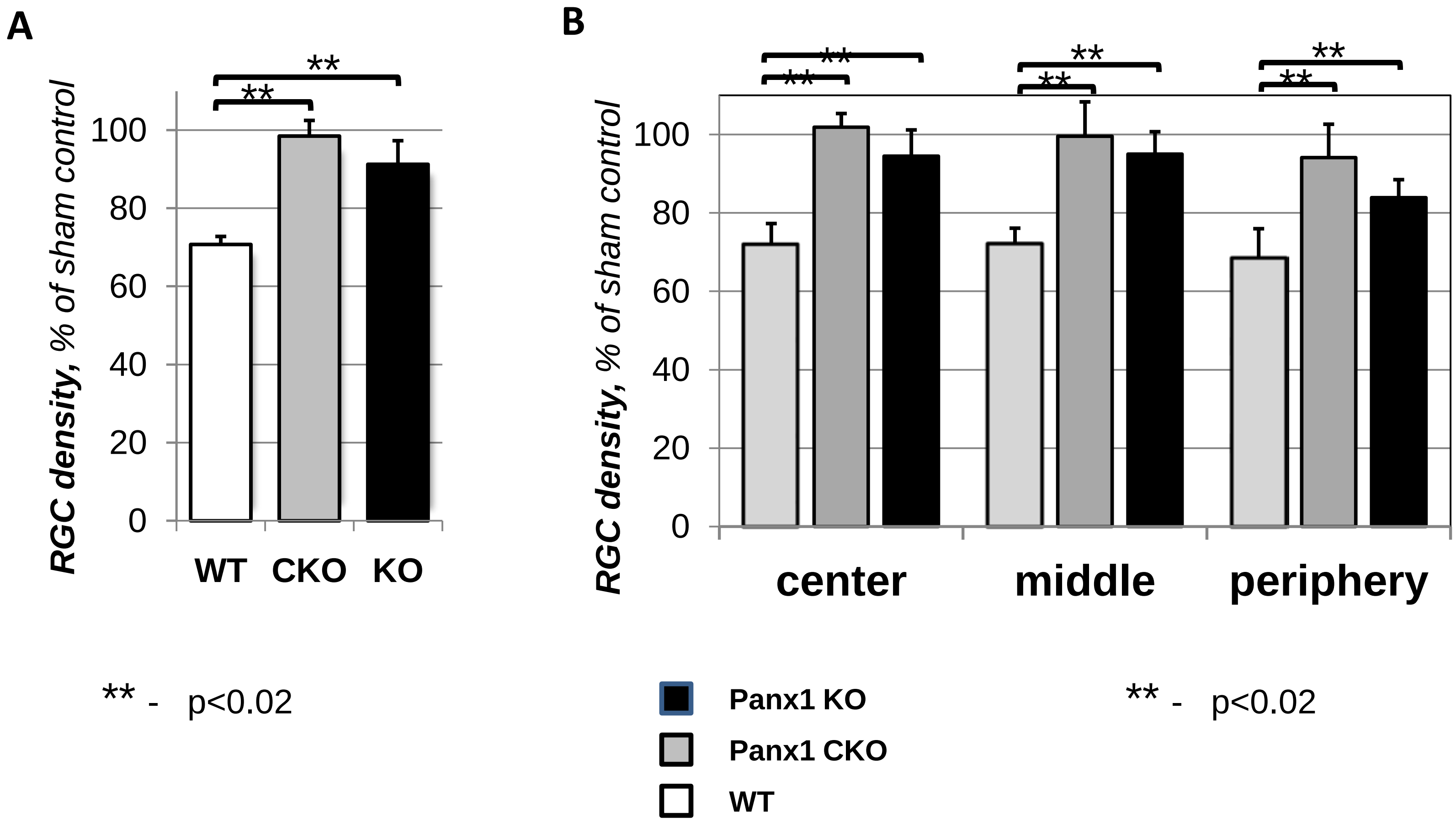


***Supplement Figure S4**. **Neuroprotection in different retinal regions of Panx1-deficient animals***
***A.*** *Averaged levels of RGC loss calculated as percentage of experimental vs. contralateral control eye* (normotensive sham-operated control ) *in Panx1 CKO (n=5), Panx1 KO (n=8) and WT (n=5) retinas exposed to IR injury; mean SEM .* ***B.*** *RGC loss in central, middle and peripheral retina; mean SEM*
