# Supplementary material for: Genetic Ablation of Pannexin1 Protects Retinal Neurons from Ischemic Injury
Source: PLoS One. 2012 Feb 23;7(2):e31991. doi: 10.1371/journal.pone.0031991 (PMC3285635; doi:10.1371/journal.pone.0031991)
Supplement: Figure S5 — Differential activation of the Il1b gene in WT vs. Panx1 KO retinal in response to IR. (PDF) [file pone.0031991.s008.pdf]

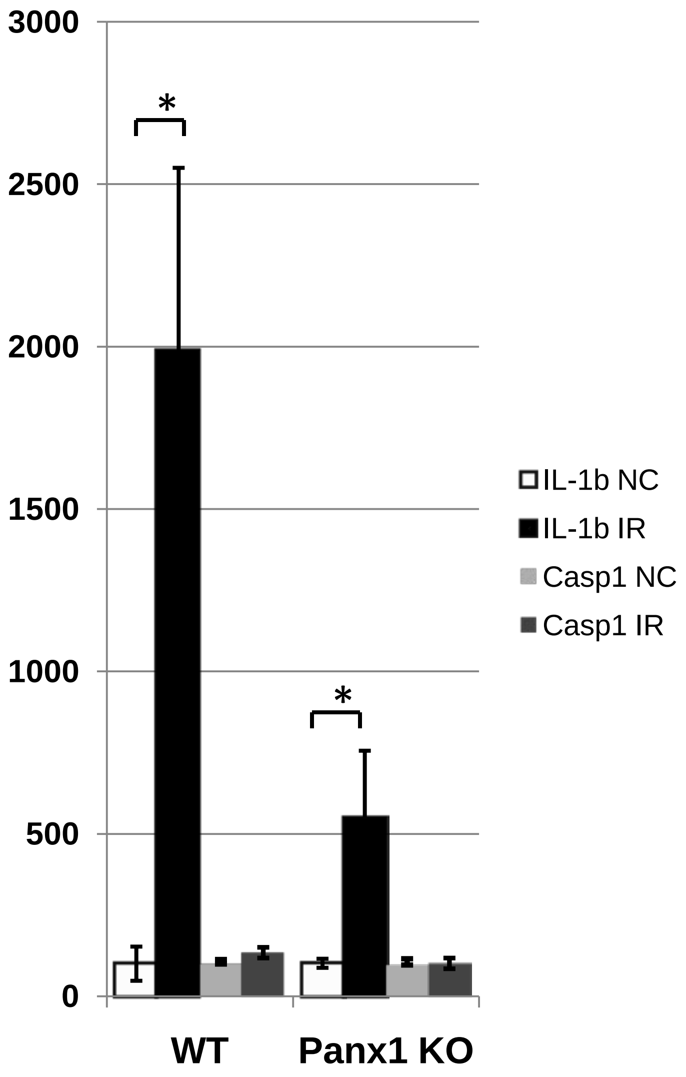

Supplement Figure S5**. Differential activation of the Il1b gene in WT vs. Panx1 KO retinal in response to IR.** Gene expression analysis in whole retina from ischemic (IR, sampled 3 hours after reperfusion) and normotensive sham-operated control (NC) eyes performed by quantitative PCR. Relative abundances of the *Il1b and Casp1* transcripts in total extracts from WT and Panx1 KO retinas normalized to the levels of β-actin transcript; data are presented as mean SD. Asterisk indicates $P<0.05$. The up-regulation of the *Il1b, but not Casp1* gene was significantly suppressed in Panx1 KO vs. WT retinas challenged by IR.
