# Supplementary material for: Genetic Ablation of Pannexin1 Protects Retinal Neurons from Ischemic Injury
Source: PLoS One. 2012 Feb 23;7(2):e31991. doi: 10.1371/journal.pone.0031991 (PMC3285635; doi:10.1371/journal.pone.0031991)
Supplement: Figure S6 — Co-localization analysis of IL-1β and caspase-1 in primary RGC after OGD. (PDF) [file pone.0031991.s009.pdf]

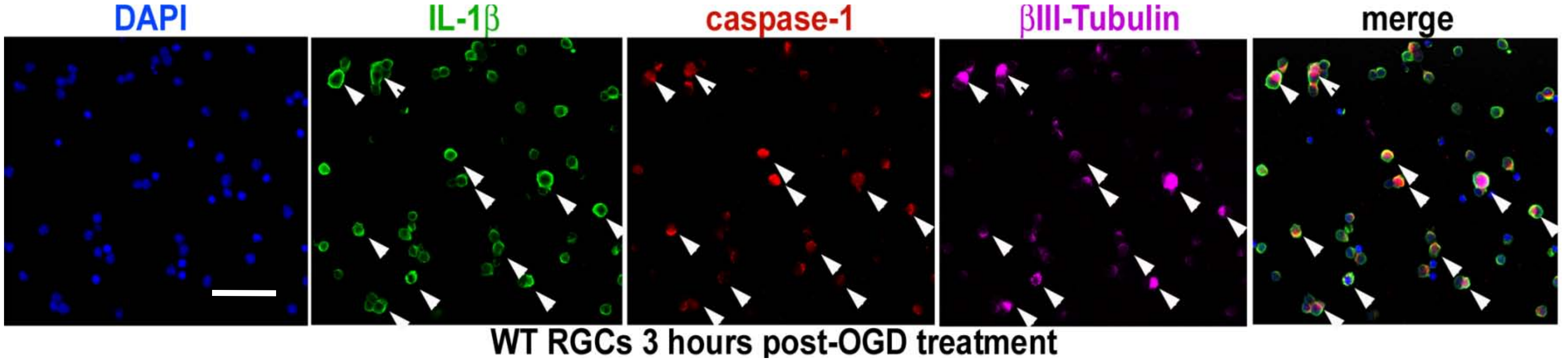

***Supplement Figure S6*. *Co-localization analysis of IL-1β and caspase-1 in primary RGCs after OGD*.**
Arrows indicate cells co-localizing staining for RGC-specific marker IIIβ–Tubulin with the labeling specific to IL-1β and caspase-1 proteins. DAPI staining for nucleic acid is in blue; scale bar, 50 μm
