# Supplementary material for: Genetic Ablation of Pannexin1 Protects Retinal Neurons from Ischemic Injury
Source: PLoS One. 2012 Feb 23;7(2):e31991. doi: 10.1371/journal.pone.0031991 (PMC3285635; doi:10.1371/journal.pone.0031991)
Supplement: Figure S7 — Co-localization analysis of inflammasome marker proteins ASC and NALP1. (PDF) [file pone.0031991.s010.pdf]

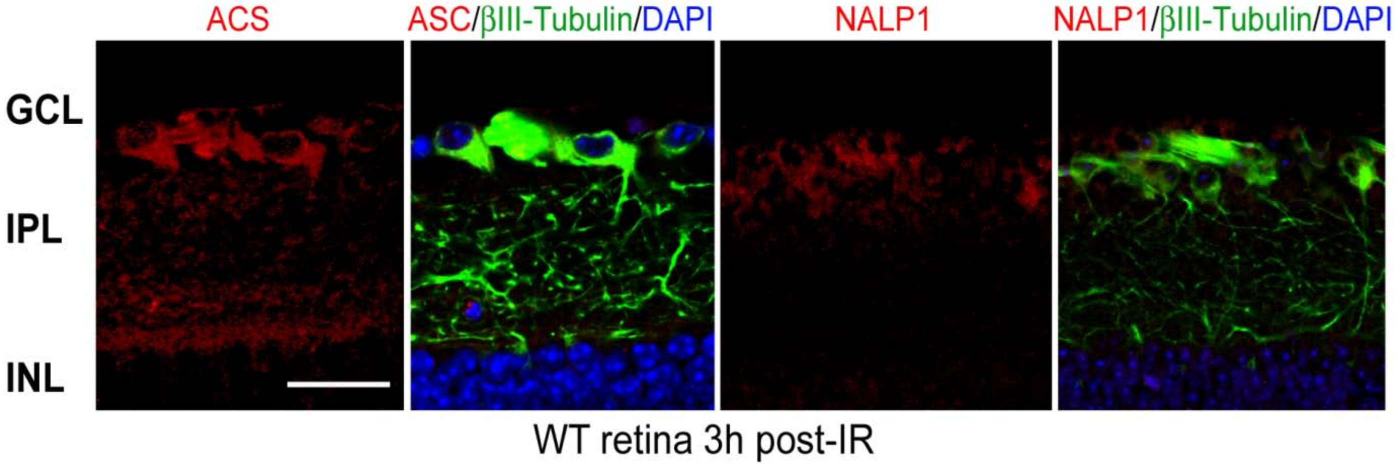

Supplement Figure S7. Co-localization analysis of inflammasome marker proteins ASC and NALP1 *in the mouse retina at 3h post-IR. Arrows indicate cells co-localizing staining for RGC-specific marker IIIβ–Tubulin with the labeling specific to ASC and NALP1 proteins. DAPI staining for nucleic acid is in blue; scale bar, 50 μm*
