# Supplementary material for: Genetic Ablation of Pannexin1 Protects Retinal Neurons from Ischemic Injury
Source: PLoS One. 2012 Feb 23;7(2):e31991. doi: 10.1371/journal.pone.0031991 (PMC3285635; doi:10.1371/journal.pone.0031991)
Supplement: Figure S8 — OGD chamber test for the pO2 kinetics. (PDF) [file pone.0031991.s011.pdf]

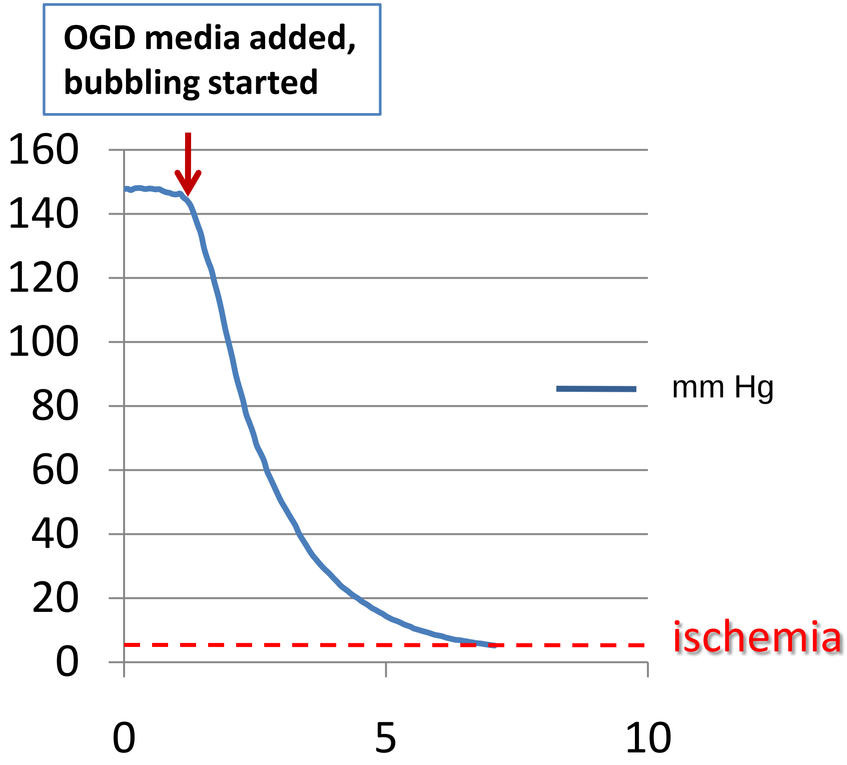

Supplement Figure S8. OGD chamber test for pO_2 kinetics. *Direct pO_2 measurements at the bottom of the microscopy ischemic chamber using direct measurement with OxyLab pO2 oxygen sensor* (Oxford Optronix Ltd.) *show averaged (n=5) curve of pO_2 reduction at 32^0C. Ischemic conditions (pO_2 <5.0 mm Hg) were achieved within 10 minutes after media change and bubbling.*
